# Event-related pupillary response-based authentication system using eye-tracker add-on augmented reality glasses for individual identification

**DOI:** 10.3389/fphys.2024.1325784

**Published:** 2024-08-13

**Authors:** Sangin Park, Jihyeon Ha, Laehyun Kim

**Affiliations:** ^1^ Industry-Academy Cooperation Team, Hanyang University, Seoul, Republic of Korea; ^2^ Bionics Research Center, Korea Institute of Science and Technology, Seoul, Republic of Korea; ^3^ Department of HY-KIST Bio-Convergence, Hanyang University, Seoul, Republic of Korea

**Keywords:** augmented reality, authentication, biometrics, event-related potential, event-related pupillary response

## Abstract

This study aimed at developing a noncontact authentication system using event-related pupillary response (ErPR) epochs in an augmented reality (AR) environment. Thirty participants were shown in a rapid serial visual presentation consisting of familiar and unknown human photographs. ErPR was compared with event-related potential (ERP). ERP and ErPR amplitudes for familiar faces were significantly larger compared with those for stranger faces. The ERP-based authentication system exhibited perfect accuracy using a linear support vector machine classifier. A quadratic discriminant analysis classifier trained using ErPR features achieved high accuracy (97%) and low false acceptance (0.03) and false rejection (0.03) rates. The correlation coefficients between ERP and ErPR amplitudes were 0.452–0.829, and the corresponding Bland–Altman plots showed a fairly good agreement between them. The ErPR-based authentication system allows noncontact authentication of persons without the burden of sensor attachment via low-cost, noninvasive, and easily implemented technology in an AR environment.

## 1 Introduction

A biometric authentication system protects significant information from people or organizations and plays an important role in the identification and authentication infrastructure (Kaongoen et al., 2017). Biometric systems are used to verify the identity of people based on their unique physiological and/or behavioral personal characteristics (Rahman et al., 2021), such as fingerprints, palm prints, face scans, iris scans, ear shapes, and vocal tract systems (Sabeti et al., 2020). Identification of people has been widely used to prevent the leakage of private information and unauthorized access of security systems in various fields such as banking, online transactions, border control, military, retail, healthcare, law enforcement, and enterprises (Rahman et al., 2022). However, many biometric traits are prone to stealing and forgery due to the advancement of related scientific techniques (Galbally et al., 2014; Ashenaei et al., 2022). Thus, exploring unique biological traits is important for biometric purposes (Rahman and Nakanishi, 2022). To enhance safety and security, many researchers have attempted to secure alternative biometric traits, such as electroencephalography (EEG) (Ashenaei et al., 2022).

In terms of robustness against hacking and forgery, EEG signals are a superior biometric approach because they have unique attributes not possessed by past biometric methods (Wang M. et al., 2021). In terms of security, EEG-based biometrics have the following advantages: 1) difficulty of duplication and theft because they are uncapturable, 2) alive biometric, and 3) unlimited replacement (Ashenaei et al., 2022). Additionally, biometric traits from a deceased (and warm) body can be used to allow authentication of security systems-; however, a dead brain cannot generate the EEG oscillations required for authentication (Norton et al., 2017). Finally, EEG signals are robust to the demand for coercive password entry because they can be easily affected by external pressures (Wu et al., 2018a). EEG-based biometric systems are primarily categorized into spontaneous EEG and evoked potentials (EPs) using signal acquisition protocols (Wang M. et al., 2021; Ashenaei et al., 2022). Resting-state EEG is a spontaneous signal that is naturally generated by the brain without any stimuli. It has been utilized in previous studies as a biometric trait (Thomas and Vinod, 2018; Kim and Kim, 2019; Maiorana, 2021) because of its flexible acquisition of signals and suitability for continuous monitoring compared with stimuli-based EPs (Thomas and Vinod, 2018). However, the stability of resting-state-based biometrics is relatively poor (Wang M. et al., 2019). In contrast, to elicit unique signals based on strict protocols, event-related potential (ERP)-based biometrics require users to pay attention to repetitive sensory stimuli (i.e., visually EPs) (Wang M. et al., 2021). ERP is a significant biometric trait that can reflect high-level neural resources such as attention and memory, which can perceive only people with knowledge of their intrinsic information (Sabeti et al., 2020). Additionally, the performance of detecting ERP components (i.e., P3) has been enhanced by improving the feature extraction and classification algorithms using deep learning methods (Wang H. et al., 2019; Wang H. et al., 2021; Wang et al., 2023a). Generally, strict protocols involving cognitive tasks are more distinctive between individuals and reproducible compared with those without tasks, but they are time-consuming (Wang et al., 2023a). Thus, an ERP-based biometric system can be a high-performance and safe authentication system, as proven in previous studies (Kaongoen et al., 2017; Chan et al., 2018; Wu et al., 2018b; Chen et al., 2020; Kasim and Tosun, 2021; Zhao et al., 2021). Although EEG signals have been studied as a unique biological trait of biometric authentication because of their advantages for safety and security (Shams et al., 2022), the attachment of a sensor on the head to acquire EEG signals is a major obstacle (Chang S. et al., 2020; Park et al., 2022). Thus, EEG biometrics, having the disadvantages of sensor attachment, inconvenience, complexity, onerous processes, and susceptibility to muscle noise, have lower usability than other biometrics (Rhee et al., 2022). While most ERP-based systems have been studied based on monitor screens, virtual or augmented reality (AR)-based smart glasses have the advantage of providing more flexibility such as freeing both hands and allowing the use of multiple devices (Uhlmann et al., 2019).

Pupil images can be easily measured using an eye-tracker device as an add-on to virtual reality or AR glasses. Pupillary rhythms, i.e., pupil size change (PSC), are reliably modulated by functional brain processes, such as cognition (Papetti et al., 2020), perception (Bradley et al., 2017), attention (Unsworth et al., 2018), memory (Stolte et al., 2020), and emotion (Cherng et al., 2020) via neural connectivity. Previous studies have demonstrated that PSC is strongly associated with neural activity in brain regions or networks that involve the locus coeruleus–norepinephrine system, dorsal attention network, posterior and anterior cingulate cortex, insular cortex, basal ganglia, and lingual gyrus (Joshi et al., 2016; Ceh et al., 2021; Groot et al., 2021; Mäki-Marttunen, 2021). Thus, PSC signals have a great potential for use in biometrics as an alternative to ERP analysis. The pupil-based biometric as a biometric trait reflecting neural activity is robust to stealing and forgery and is simple and convenient compared with ERP in terms of usability. Previous studies have reported a significant correlation between ERP components and PSC (Widmann et al., 2018; Schütte et al., 2021; Selezneva and Wetzel, 2022). Several studies have also attempted to directly compare ERP and PSC epochs and have reported mutual similarities and replaceability (Park et al., 2019; Dahl et al., 2020; Park et al., 2022). The PSC epoch caused by a target stimulus is called event-related pupillary response (ErPR).

The aim of this study is to develop an ErPR-based biometric authentication system using a rapid serial visual presentation (RSVP) paradigm with human photograph stimuli in AR glasses. The RSVP paradigm, which can present a large number of stimuli in a short time, can elicit strong EPs caused by a stimulus paradigm consisting of targets and nontargets (Acqualagna and Blankertz, 2013), and has been utilized in many visual-based brain-computer interface studies (Zhang H. et al., 2022; Wang et al., 2023b; Wang et al., 2024). Additionally, numerous ERP-based studies have reported that ERP components (i.e., N2, P3, N4, and P6) are significant features for distinguishing familiar from stranger human faces (Hanso et al., 2010; Huang et al., 2017; Chang W. et al., 2020). We elicited stronger subject-specific ErPR from pupil images and ERP from EEG signals using our RSVP paradigm, which included familiar (target) or stranger (nontarget) human photographs. Two biometric traits were compared using accuracy, area under the receiver operating characteristics curve (AUC), false rejection rate (FRR), and false acceptance rate (FAR). Detailed information on the proposed biometric method and results are described in the following sections.

## 2 Materials and methods

### 2.1 Subjects

Thirty healthy volunteers (15 men and 15 women), aged between 22 and 33 years (mean age, 27.20 ± 3.34 years) participated in this experiment. All the participants had normal or corrected-to-normal vision (i.e., over 0.8) and were right-handed. Each participant participated voluntarily and was paid 30,000 KRW. None of them had any history of serious medical or psychological illnesses. Written informed consent was obtained from all participants, and they were notified of the restrictions and requirements. All the experimental protocols were approved by the Ethics Committee of the Korea Institute of Science and Technology, Seoul, South Korea (approval number: 2021-012).

### 2.2 Experimental procedure and stimuli

The participants were required to provide photographs of familiar faces of their family or friends of the same gender. In total, 300 photographs of familiar faces were collected from 30 participants, with 10 photographs per participant. For each subject, ten photographs were randomly presented throughout the entire experiment as the target stimuli. In all the trials, a total of 900 photographs of random Korean individuals (450 men and 450 women) were collected and used as nontarget stimuli. Repeated exposure to a stranger’s face (i.e., familiarization) may induce ERP patterns similar to those of a familiar face (Campbell and Tanaka, 2021). Thus, all nontarget stimuli were presented only once without duplication, and the order was counterbalanced. To minimize the effects of gender and race on ERP (Ito and Urland, 2005), all photographs used in this experiment consisted of a Korean person, and photographs of the same gender as the subjects were presented as both the target and nontarget stimuli. All the photographs were set up to be the same orientation and size.

**FIGURE 1:**
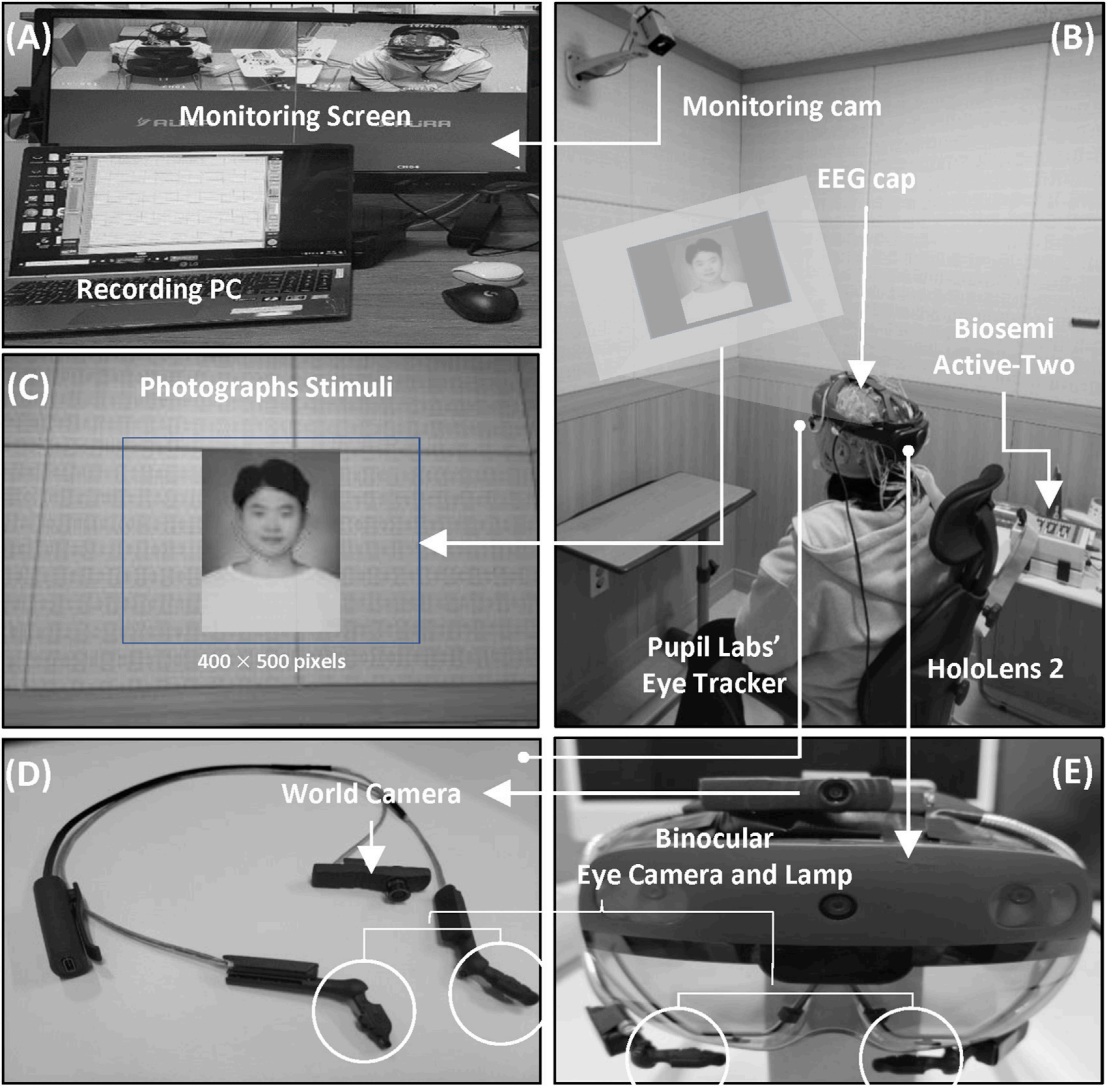
Overview of the experimental setup. **(A)** Experimental management and monitoring. **(B)** Experimental environment. **(C)** Example of an AR stimulus. **(D)** Pupil Labs’ AR binocular eye-tracker add-on AR device. **(E)** AR device (HoloLens 2).

Each participant wore a Microsoft HoloLens 2 (Microsoft Corp., Redmond, WA, United States) in an electrically shielded room and sat in a comfortable armchair. An electrically shielded room was used to minimize the risk of external interference during the measurement of EEG signals and to increase the concentration of the subjects. Participants were required to perform an ERP task, and EEG signals and pupil images were measured during the task. The overall process of the experiment was recorded using a monitoring camera, as shown in Figure 1.

**FIGURE 2:**
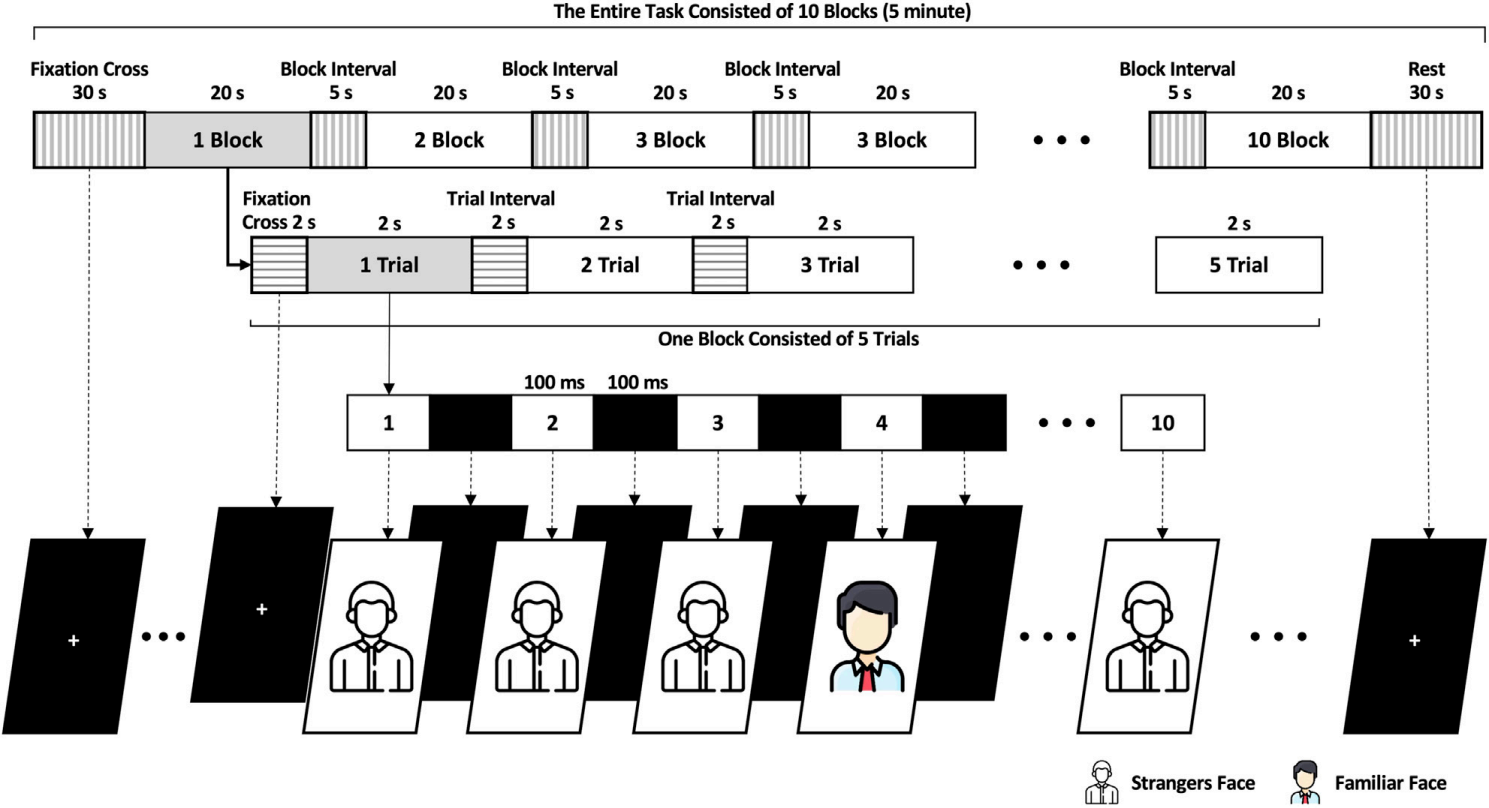
Overview and temporal scheme of the ERP task.

As Figure 2 shows, the participants executed the ERP task for approximately 5 min. The ERP task began by displaying a cross on the AR screen’s center for 2 s followed by ten face photographs, each shown for 2 s. The ten face photographs were arranged randomly, with only one featuring as the target familiar face selected randomly from a database of the subject’s photographs. The other nine photographs presented random strangers’ faces as non-targets. Each photographic stimulus was presented center screen for 100 ms, and a block screen was shown for 100 ms between photographs (i.e., the distance between two subsequent stimuli is 200 ms). One trial comprised ten face photograph stimuli and lasted 2 seconds. One block comprised five trials, each separated by a 2-s interval, totaling 20 s. The entire experiment consisted of 50 trials (ten blocks) with an inter-block interval of 5 s resulting in a total duration of 245 s. Before and after the experiment, there were periods of preparation and relaxation, each lasting for 30 s. The stimuli displayed at the center of the screen and the size of the photographs, including the black screen were 400 × 500 pixels. The stimuli were presented on a transparent display on an AR headset (MS Microsoft HoloLens 2™ Microsoft Corp., Redmond, WA, United States). The event triggers for the stimuli were synchronized with the EEG and ErPR data through User Datagram Protocol communication. When the stimuli began on the AR device, the recording software for EEG and ErPR on the laptop received UDP packets from the AR device. The data was then saved with the specific time corresponding to when the UDP packet was received at the start of the stimuli.

### 2.3 Data acquisition and signal processing

A 64-channel BioSemi ActiveTwo system (BioSemi BV, WG-Plein, Amsterdam, Netherlands) was used to acquire EEG signals from the participants at a sampling rate of 2048 Hz with an EEG cap of active Ag/AgCl electrodes through a conductive water-based gel (CG04 Saline Base Signa Gel, Parker Laboratories Inc., Fairfield, NJ, United States) arranged in an international 10–20 system (ground: common mode sense; reference: driven right-leg electrodes). The electrode impedance between the measurement and ground electrodes was maintained below 10 kΩ. To avoid contaminating meaningful patterns of ERP, muscle artifacts of oculomotor were removed from the raw EEG signals using independent component analysis based on visual inspection (Katus et al., 2020; Li et al., 2020). Pupil images were taken on Pupil Labs’ AR binocular eye-tracker (Pupil Labs, Berlin, BB, Germany) add-ons for the HoloLens 2 at a sampling rate of 200 fps with a resolution of 192 × 192 pixels using Pupil Core software (Pupil Labs, Berlin, BB, Germany). This software provides data related to eye movement, including gaze position and pupil diameter. A previous study confirmed that pupil size increases as illumination decreases. Under five different lighting conditions, the average pupil diameter measured 3.5 mm at 550 lx, 4.2 mm at 350 lx, 5.2 mm at 150 lx, 5.03 mm at 40 lx, and 5.4 mm at 2 lx. The pupil size significantly increased when the illumination changed from 550 to 150 lx; however, lighting conditions of 150, 40, and 2 lx minimally impacted the changes in pupil size (Maqsood and Schumacher, 2017). To minimize the effect on pupil size caused by a significant change in ambient light, ambient light in the electrically shielded room was controlled at 150 lx or less (Park et al., 2021; Park et al., 2022). During the experiment, the ambient light was measured using a Visible Light SD Card Logger (Sper Scientific Meters Ltd., Scottsdale, AZ, United States) at a 1 Hz sampling rate in both the experiment room (105.47 ± 2.22 lx) and AR device (30.70 ± 7.75 lx).

### 2.4 Data processing

**FIGURE 3:**
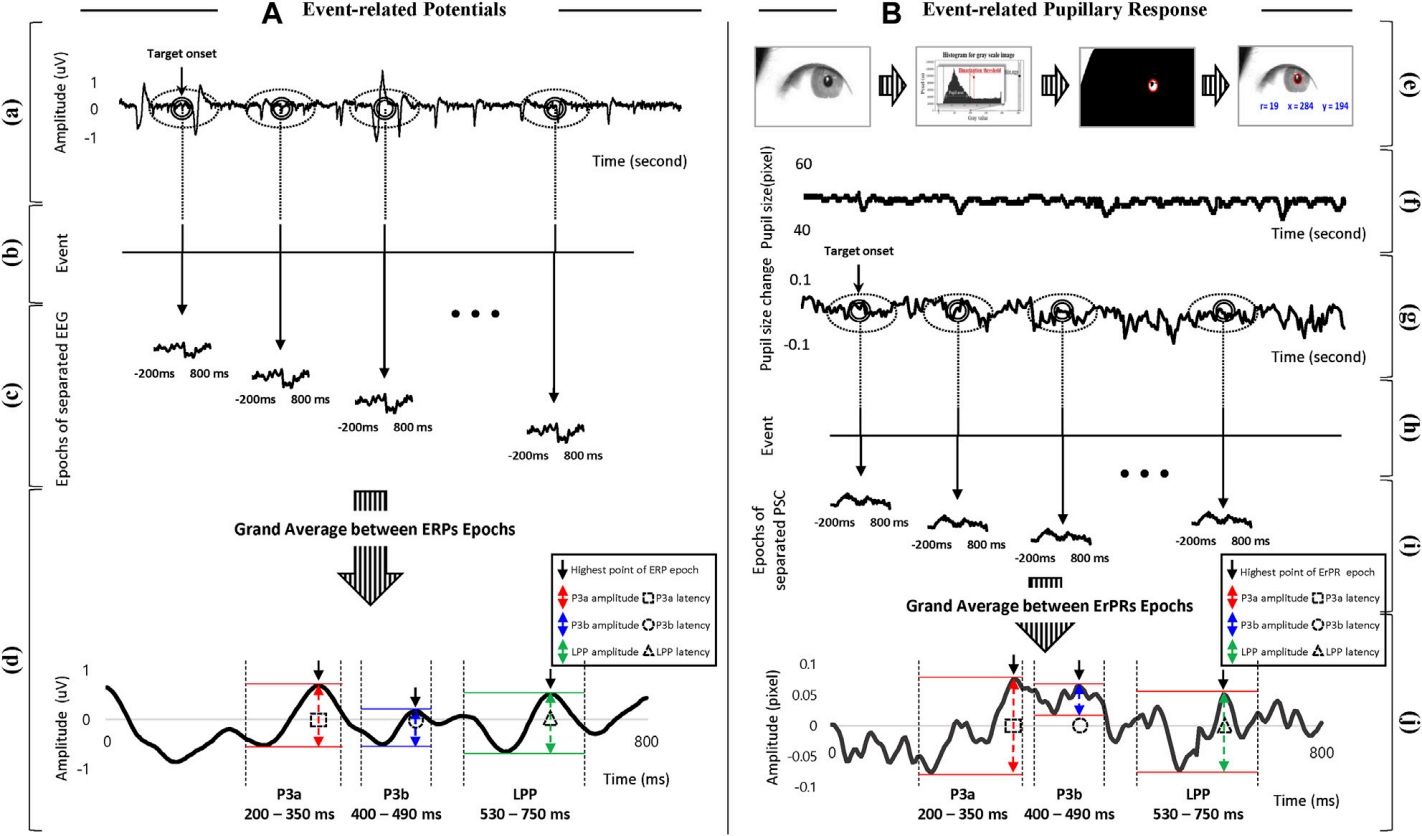
Overview of signal processing with definition of features in **(A)** ERP and **(B)** ErPR. (a) EEG raw signals. (b), (h) Time log of target onset. (c) Epochs of separated EEG signals based on target onset. (d) Grand averages for all separated EEG epochs, and definition of amplitude and latency in ERP including time windows of P3a, P3b, and LPP. (e) Procedure for detecting pupil area. (f) Signals of pupil diameter (raw data). (g) Signals of PSC calculated from frame difference. (i) Epochs of separated PSC data based on target onset. (j) Grand averages for all separated PSC epochs, and definition of amplitude and latency in ErPR including time windows of P3a, P3b, and LPP.

The procedure for signal processing in the ERP was as follows: 1) The EEG signals were down sampled from 2048 to 200 Hz, and then filtered using a fourth-order Butterworth band-pass filter (0.1–50 Hz). 2) The filtered EEG data were segmented into EEG epochs with lengths of 800 ms from 200 ms before the onset of each stimulus to represent the stimulus. 3) All EEG epochs were corrected at baseline by averaging EEG epochs lasting 800 ms using 200 ms of data before target onset (Mitchell et al., 2016). 4) EEG epochs in all trials were averaged with lengths of 800 ms. 5) The amplitude and latency were defined by calculating difference values in amplitude between the lowest and highest points and the time value of the highest point in the ERP epoch, respectively, within a time window of 200–750 ms (Kaongoen et al., 2017), as shown in Figure 3A. The time windows were divided into P3a (200–350 ms), P3b (400–490 ms), and LPP (530–750 ms) (Takeda et al., 2014; Causse et al., 2016). Amplitude and latency were extracted from three time domains and four brain regions (Fz, Cz, Pz, and Oz electrodes) respectively. All signal processing and data analyses were performed using EEGlab, which is a MATLAB toolbox (R2020b; MathWorks Inc., Natick, MA, United States).

The procedure for signal processing in ErPR was as follows: 1) The pupil diameter was obtained from data offered by the Pupil Core software at a sampling rate 200 fps, and data of the dominant eye for each subject were used for analysis. The dominant eye of each individual was identified using the hole-in-the-card test (Li et al., 2010). 2) PSCs were calculated using the difference between the frames for each pupil diameter. 3) All PSC epochs were corrected to a baseline by the averaged PSC epochs lasting 800 ms using 200 ms of data before the target onset. 4) The PSC epochs in all the trials were averaged with lengths of 800 ms, and the average PSC epoch was defined as ErPR. 5) The amplitude and latency of the ErPR epoch were defined by calculating the difference between the lowest and highest PSC points and the time value of the highest PSC point, respectively, within the time windows of P3a (200–350 ms), P3b (400–490 ms), and the LPP (530–750 ms), consistent with the ERP epochs, as shown in Figure 3B. All signal processing was performed using the MATLAB signal processing toolbox (R2020b, MathWorks Inc., Natick, MA, United States).

### 2.5 Statistical analysis and classification

This study has a design within subject, wherein two stimuli such as target (i.e., familiar face) and nontarget photograph (i.e., stranger face) are tested on each test subject. Thus, for the statistical analysis, a paired-samples *t*-test was used to compare the responses of individual participants between target and nontarget stimuli based on the Shapiro–Wilk normality test (*p* > 0.05). The recommended total sample size, calculated using G*Power software (ver. 3.1.9.7; Heinrich-Heine-Universität Düsseldorf, Düsseldorf, Germany), was 54 samples (α = 0.01, 1 – β = 0.95, effect size = 0.5), and this study (i.e., 60 samples size) satisfied the recommended sample size from G*Power (Faul et al., 2007). The significant level to test hypotheses was controlled by the number of individual hypotheses (i.e., α = 0.05∕n) to resolve the problem of type-I errors by multiple comparisons (Jafari and Ansari-Pour, 2019) as follows: the statistically significant level was set to 0.0017 (i.e., α = 0.05/30; 24 ERP and six ErPR features). Moreover, this study confirmed the practical significance of using an effect size of Cohen’s *d*, with the standard values of 0.20, 0.50, and 0.80 regarded as small, medium, and large, respectively (Huck et al., 1974). All statistical analyses were conducted using IBM SPSS Statistics for Windows (SPSS Corp., Armonk, NY, United States).

To determine the best classification algorithm for the two conditions (ERP and ErPR features), two machine-learning algorithms were used: 1) linear support vector machine (LSVM), 2) quadratic discriminant analysis (QDA), 3) Naïve Bayes (NB), 4) logistic regression (LR), and 5) radial basis function support vector machine (RBF-SVM). Optimization results for each classification method were obtained through five-fold cross-validation using “scikit-learn” (ver. 0.24.2) of Python (ver. 3.6.9). To assess practical classification performance, we reduced the number of trials while maintaining a 1:9 ratio between familiar and unfamiliar stimuli. To extract the features, we computed the averaged ERP and ErPR epochs over the familiar and unfamiliar stimuli trials. From the averaged ERP epochs, we extracted 12 features, which comprised four channels (Fz, Cz, Pz, and Oz) and three indicators (P3a, P3b, and LPP) for the ERP features. From the averaged ErPR epochs, we also extracted three features including three indicators (P3a, P3b, and LPP) for ErPR features. The structures of the features for each condition were: 1) 60 samples (30 subjects and two conditions) × 12 ERP features and 2) 60 samples (30 subjects and two conditions) × three ErPR features. Accuracy refers to the average accuracy of the five-fold cross-validation. Moreover, the FAR, FRR, and AUC were evaluated.

The accuracy was calculated using the proportion of the total number of correct predictions, as shown in Equation 1.

$$Accuracy\;\left(\%\right)=\frac{TP+TN}{TP+FN+TN+FP}\times 100$$ (1)

A true positive (TP) is a correctly classified target. A false negative (FN) is an incorrectly classified target. A true negative (TN) is a correctly classified nontarget. A false positive (FP) is an incorrectly classified nontarget. FAR is the proportion of identification instances in which unauthorized persons are incorrectly accepted, and FRR is that in which authorized persons are incorrectly rejected. These values were calculated using Equation 2.

$$FAR=\frac{FP}{FP+TN},FRR=\frac{FN}{FN+TP}$$ (2)

## 3 Results

### 3.1 Averaged plot of ERP and ErPR epochs from all subjects

**FIGURE 4:**
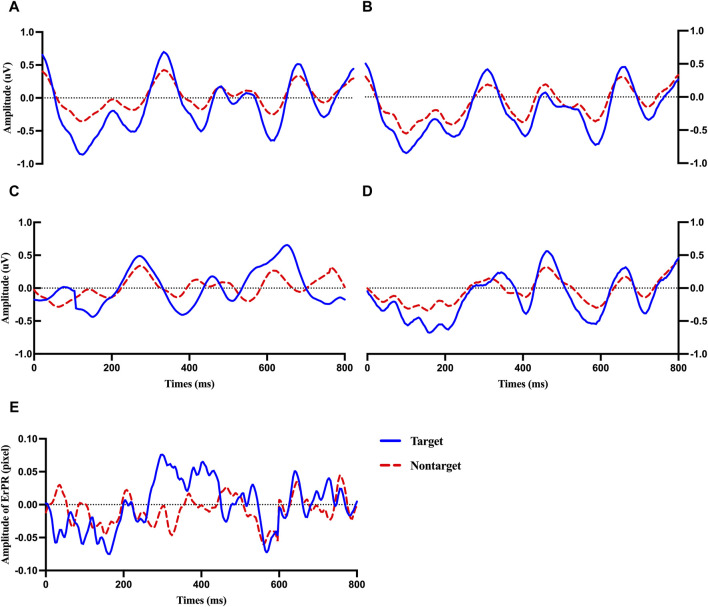
Averaged plot of ERP at Fz **(A)**, Cz **(B)**, Pz **(C)**, and Oz **(D)** and ErPR **(E)** from all subjects for target and nontarget stimuli.

Figure 4 shows the average ERP plot of each channel (Fz, Cz, Pz, and Oz) and the ErRPs for the target and non-target stimuli. Each average plot includes 50 target epochs and 450 nontarget epochs. The solid and dotted lines indicate the ERP or ErPR epochs of the participants after being presented with the target and nontarget stimuli, respectively. The evoked positive potentials within P3a (200–350 ms), P3b (400–490 ms), and LPP (530–750 ms) were clearly observed in both the target and nontarget ERP epochs in the Fz, Cz, Pz, and Oz regions. The increasing ERP amplitude trend was observed for the target stimuli from P3a, P3b, and LPP compared with the nontarget stimuli in all electrode channels. No clear difference was observed in EEG latency. These trends of amplitude and latency of ERP are similar to those observed in the ErPR epoch.

### 3.2 Amplitude and latency of ERP epoch

**FIGURE 5:**
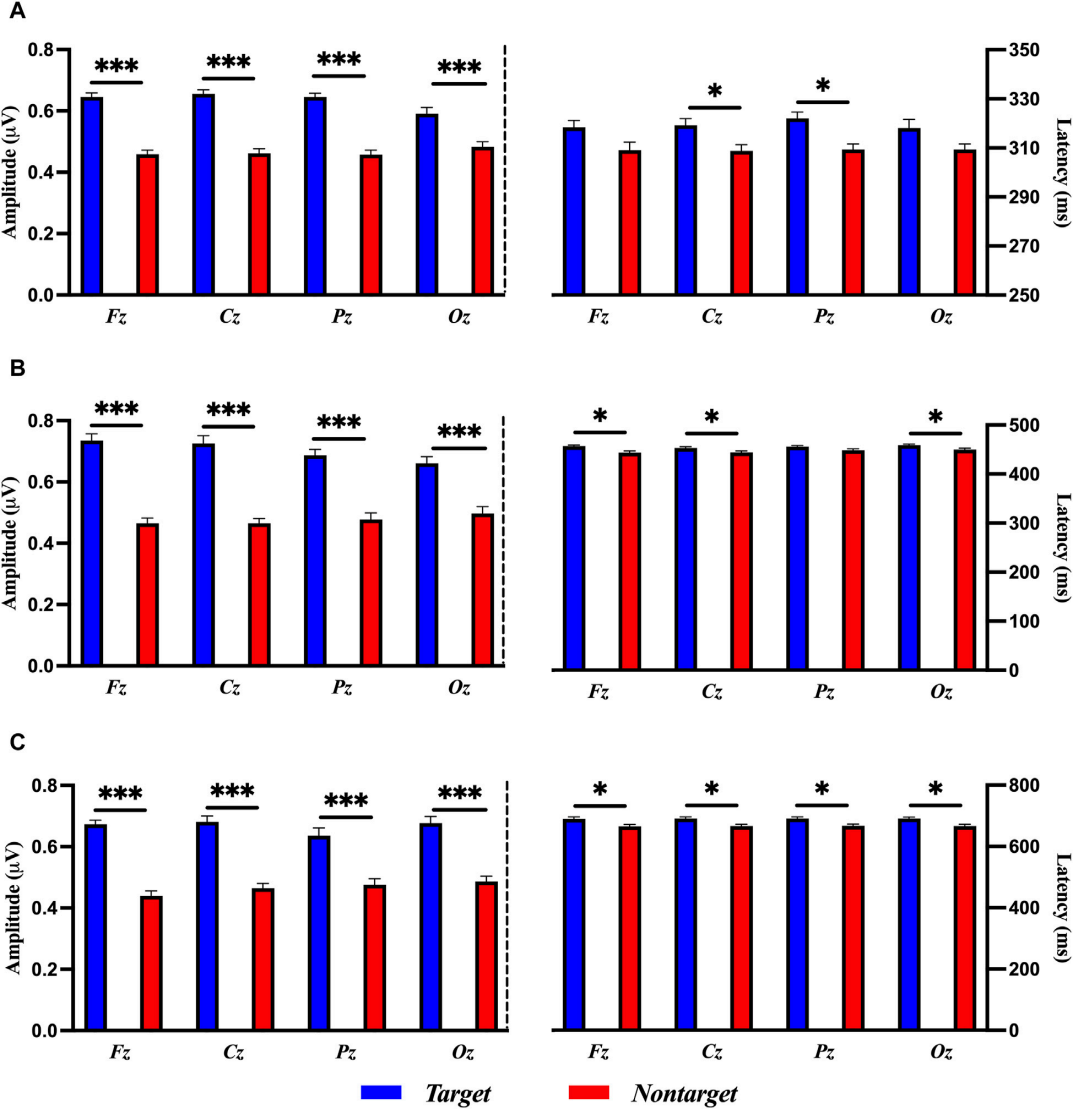
Comparisons of ERP amplitude and latency for target and nontarget stimuli in **(A)** P3a, **(B)** P3b, and **(C)** LPP with a paired-samples *t*-test. The error bars show the standard error in each condition (*, *p* < 0.05, ***, *p* < 0.001).

Figure 5 shows the results of a paired-sample *t*-test between the target and nontarget stimuli in terms of the amplitude and latency of ERP. The amplitude of ERP of target stimuli in P3a (200–350 ms) component was significantly larger than those of nontarget stimuli in Fz [*t* (58) = 8.445, *p* < 0.001, Cohen’s *d* = 2.527], Cz [*t* (58) = 8.637, *p* < 0.001, Cohen’s *d* = 2.599], Pz [*t* (58) = 9.595, *p* < 0.001, Cohen’s *d* = 2.667], and Oz [*t* (58) = 3.789, *p* < 0.001, Cohen’s *d* = 1.036]. The amplitude of ERP of target stimuli in P3b (400–490 ms) component was significantly larger than those of nontarget stimuli in Fz [*t* (58) = 10.027, *p* < 0.001, Cohen’s *d* = 2.509], Cz [*t* (58) = 9.243, *p* < 0.001, Cohen’s *d* = 2.306], Pz [*t* (58) = 6.497, *p* < 0.001, Cohen’s *d* = 1.925], and Oz [*t* (58) = 4.786, *p* < 0.001, Cohen’s *d* = 1.353]. The amplitude of ERP of target stimuli in LPP (530–750 ms) component was significantly larger than those found in nontarget stimuli in Fz [*t* (58) = 12.661, *p* < 0.001, Cohen’s *d* = 2.967], Cz [*t* (58) = 10.470, *p* < 0.001, Cohen’s *d* = 2.298], Pz [*t* (58) = 4.367, *p* < 0.001, Cohen’s *d* = 1.290], and Oz [*t* (58) = 6.830, *p* < 0.001, Cohen’s *d* = 1.809].

The latency of ERP in P3a, P3b, and LPP revealed that there was no significant difference between target and nontarget stimuli in all electrode sites as follows: P3a in Fz [*t* (58) = 1.752, *p* = 0.0903], Cz [*t* (58) = 2.341, *p* = 0.0263, adjusted by the Bonferroni correction], Pz [*t* (58) = 3.201, *p* = 0.0033, adjusted by the Bonferroni correction], and Oz [*t* (58) = 6.830, *p* = 0.0678]; P3b in Fz [*t* (58) = 2.905, *p* = 0.0070, adjusted by the Bonferroni correction], Cz [*t* (58) = 2.419, *p* = 0.0220, adjusted by the Bonferroni correction], Pz [*t* (58) = 1.990, *p* = 0.0561], and Oz [*t* (58) = 2.494, *p* = 0.0186, adjusted by the Bonferroni correction]; LPP in Fz [*t* (58) = 2.731, *p* = 0.0106, adjusted by the Bonferroni correction], Cz [*t* (58) = 2.819, *p* = 0.0086, adjusted by the Bonferroni correction], Pz [*t* (58) = 3.141, *p* = 0.0039, adjusted by the Bonferroni correction], and Oz [*t* (58) = 2.875, *p* = 0.0075, adjusted by the Bonferroni correction], as shown in Figure 5.

### 3.3 Amplitude and latency of ErPR epoch

**FIGURE 6:**
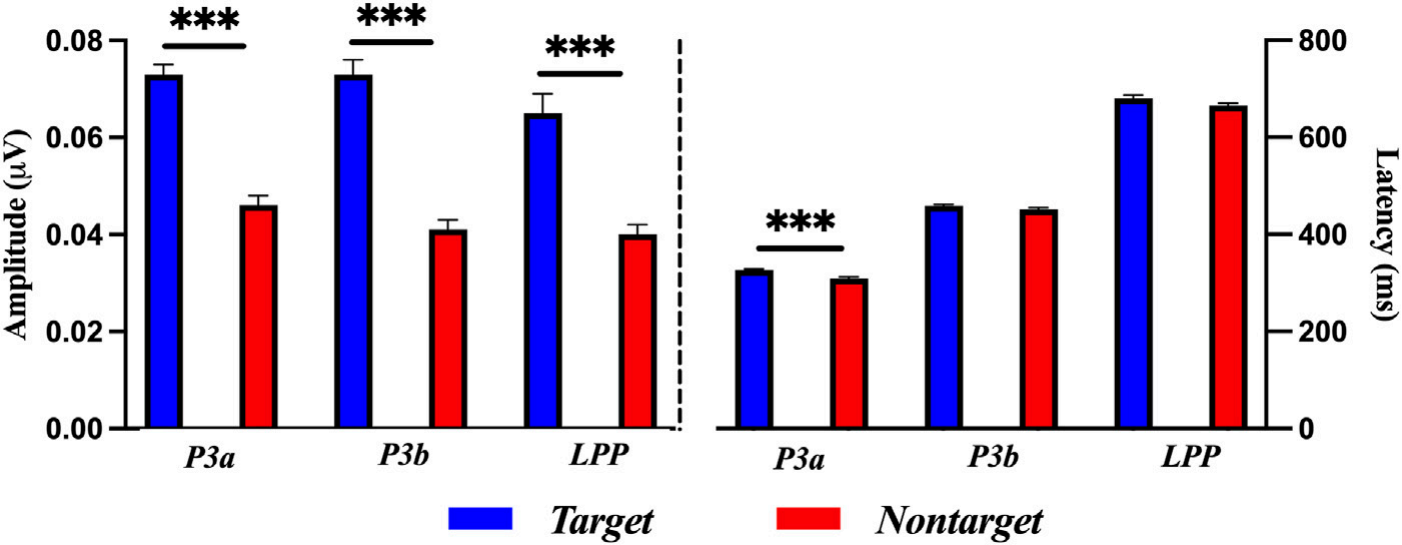
Comparisons of ErPR amplitude and latency for target and nontarget stimuli in each P3a, P3b, and LPP with a paired-samples *t*-test. The error bars show the standard error in each condition (***, *p* < 0.001).

Figure 6 shows the results of a paired-sample *t*-test for target and nontarget stimuli in amplitude and latency of ErPR. The amplitude of the ErPR of the target stimuli was significantly larger than those of the nontarget stimuli in the P3a [*t* (58) = 7.275, *p* < 0.001, Cohen’s *d* = 2.242], P3b [*t* (58) = 8.165, *p* < 0.001, Cohen’s *d* = 2.235], and LPP [*t* (58) = 5.472, *p* < 0.001, Cohen’s *d* = 1.610]. The latency of the ErPR of target stimuli was significantly delayed compared to that of nontarget stimuli at P3a [*t* (58) = 4.298, *p* < 0.001, Cohen’s *d* = 1.140]. No significant differences were found between the ErPR latencies for the target and nontarget stimuli in the P3b [*t* (58) = 1.861, *p* = 0.0729] and LPP [*t* (58) = 1.685, *p* = 0.1028].

### 3.4 Correlation and Bland–Altman plot among ERP and ErPR

**FIGURE 7:**
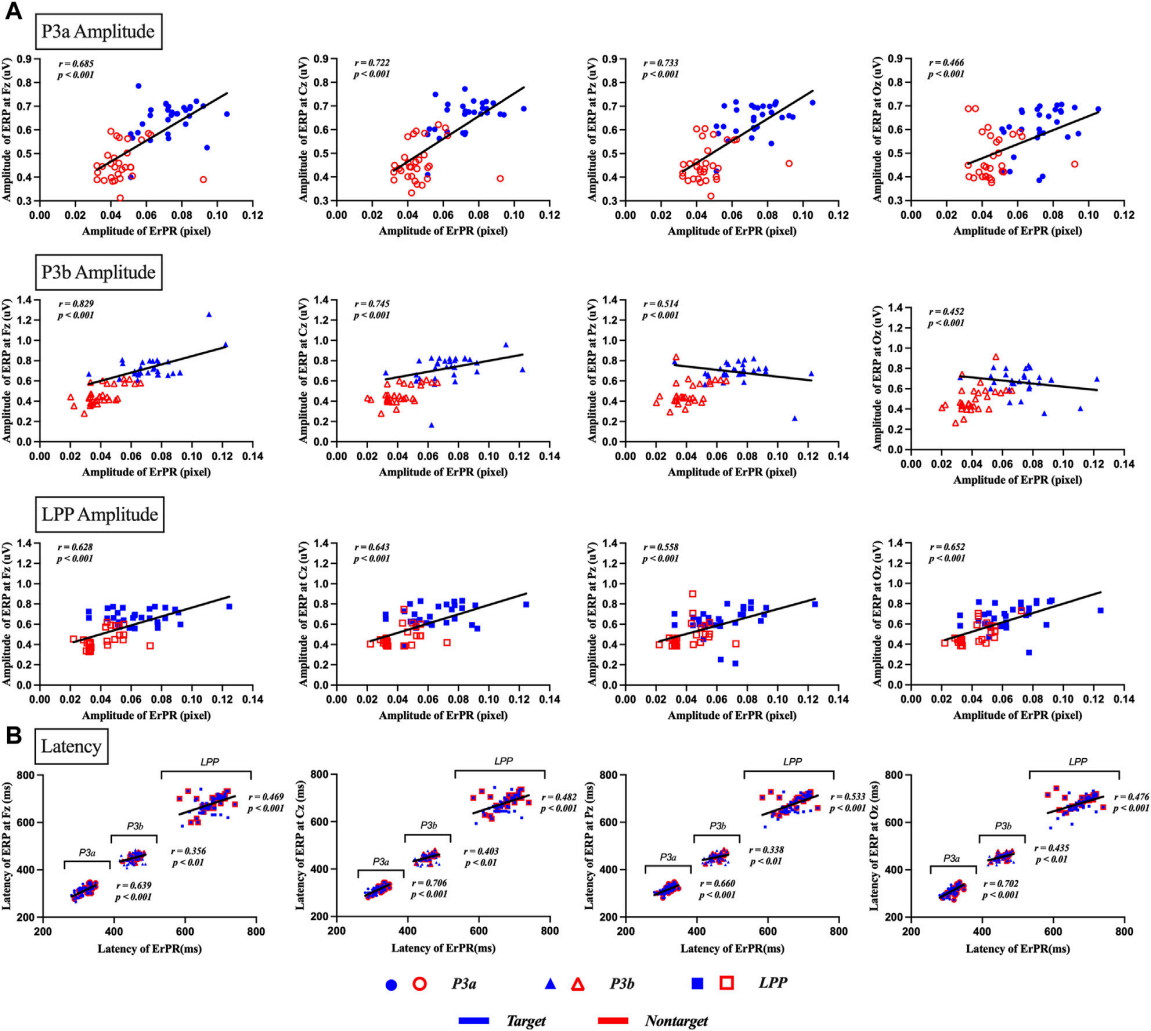
Results of correlation analysis between ERP and ErPR amplitude and latency for target and nontarget stimuli in P3a, P3b, and LPP. **(A)** depicts the correlation of amplitude between ERP and ErPR, with each row representing the amplitude of P3a, P3b, and LPP as indicated by the subtitles in the boxes. Each column shows the results for different channels: Fz, Cz, Pz, and Oz. **(B)** depicts the correlation for latency in ERP and ErPR, with each plot showing the results for P3a latency, P3b latency, and LPP latency. Each column represents the results for the channels Fz, Cz, Pz, and Oz. Significant findings were plotted as linear regression lines (*p* < 0.01, *p* < 0.001).

The Pearson correlation coefficient between the ERP and ErPR amplitudes was statistically significant. (1) P3a at Fz (*r* = 0.685, *p* < 0.001), Cz (*r* = 0.722, *p* < 0.001), Pz (*r* = 0.733, *p* < 0.001), and Oz (*r* = 0.466, *p* < 0.001). (2) P3b at Fz (*r* = 0.829, *p* < 0.001), Cz (*r* = 0.745, *p* < 0.001), Pz (*r* = 0.514, *p* < 0.001), and Oz (*r* = 0.452, *p* < 0.001). (3) LPP at Fz (*r* = 0.628, *p* < 0.001), Cz (*r* = 0.643, *p* < 0.001), Pz (*r* = 0.558, *p* < 0.001), and Oz (*r* = 0.652, *p* < 0.001). The Pearson correlation coefficient between the ERP and ErPR latency was found to be statistically significant. (1) P3a at Fz (*r* = 0.639, *p* < 0.001), Cz (*r* = 0.706, *p* < 0.001), Pz (*r* = 0.660, *p* < 0.001), and Oz (*r* = 0.702, *p* < 0.001). (2) P3b at Fz (*r* = 0.356, *p* < 0.01), Cz (*r* = 0.403, *p* < 0.01), Pz (*r* = 0.338, *p* < 0.01), and Oz (*r* = 0.435, *p* < 0.001). (3) LPP at Fz (*r* = 0.469, *p* < 0.001), Cz (*r* = 0.482, *p* < 0.001), Pz (*r* = 0.533, *p* < 0.001), and Oz (*r* = 0.476, *p* < 0.001), as shown in Figure 7.

**FIGURE 8:**
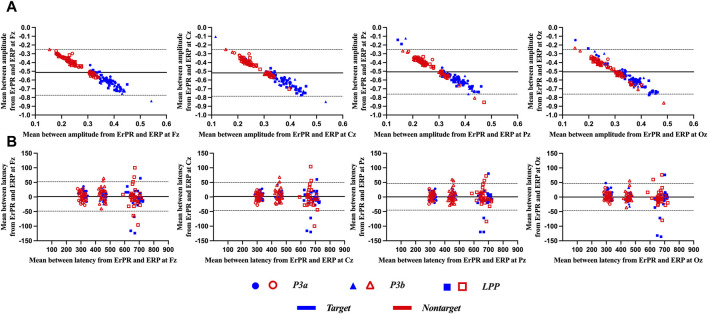
Representative Bland–Altman plots for **(A)** amplitude and **(B)** latency from ERP and ErPR epochs. The solid central line in each plot represents the mean difference between the two variables, and the upper and lower dotted lines represent the limit of the 95% confidence interval ( $\bar{d}\pm 1.96\text{ SD}$ , *n* = 180).

Figure 8 shows the results of the Bland–Altman plot between the ERP and ErPR features. It was used to visualize the differences in measurements between the two different variables. The *x*- and *y*-axes of the plot display the mean values of two variables and difference between the two variables, respectively, and involved the following three lines: the mean difference between two variables (i.e., $\bar{d}$ ), the upper (i.e., $\bar{d}+1.96*\text{SD}$ ), and lower (i.e., $\bar{d}-1.96*\text{SD}$ ) limit of the 95% confidence interval for the mean difference. If all measured values from the two variables are within $\bar{d}\pm 1.96\text{ SD}$ , it is interpreted as a good agreement between the two measurements (Martin Bland and Altman, 1986). Depending on the distribution of measured values, the interpretation of the Bland–Altman plot is categorized into “good agreement,” “fairly good agreement,” and “poor agreement,” (Gardener et al., 2020). From the results of the Bland–Atman plot, most amplitude features from ERP and ErPR were within the 95% limits, except for a few measurements, interpreted as fairly good agreement. For latency, relatively large measurements were located outside the 95% limits, interpreted as poor agreement.

### 3.5 Classification

To distinguish between the target and nontarget stimuli, the ERP and ErPR classification performances were compared. We used the twelve features for the ERP condition and the three features for the ErPR condition, which were the amplitudes of the P3a, P3b, and LPP components based on statistical significance. LSVM and RBF-SVM were the superior classifier with 100% accuracy (versus 98% with QDA) when using ERP features, while QDA with 97% accuracy outperformed LSVM (83% accuracy) on ErPR features. Overall, the classification accuracy using the ERP features was 3% greater than that with the ErPR features. Details of the classification results are listed in Table 1. Additionally, a permutation test was conducted to determine the accuracy and generalization ability of five classifiers (repeated 10,000 times), and all classifiers for both the ERP and ErPR datasets were significant (*p* < 0.0001) in the permutation test.

**TABLE 1:** Results of classification using LSVM, QDA, NB, LR, and RBF-SVM (five-fold cross-validation) among target and nontarget epochs for ERP and ErPR (N = 50).

	Classifier	Accuracy (%)	AUC	FAR	FRR
ERP	LSVM	100.0	1	0	0
	QDA	98.0	0.99	0	0.03
	NB	96.7	0.99	0.03	0.03
	LR	95.0	0.99	0	0.03
	RBF-SVM	100.0	1	0	0
ErPR	LSVM	83.0	0.99	0	0.33
	QDA	97.0	0.99	0.03	0.03
	NB	93.3	0.97	0.1	0.03
	LR	98.0	0.92	0.07	0.07
	RBF-SVM	96.7	0.99	0.06	0

LSVM, linear support vector machine; QDA, quadratic discriminant analysis; NB, Naïve Bayes; LR, logistic regression; RBF-SVM, radial basis function support vector machine; AUC, area under the receiver operating characteristic curve; FAR, false acceptance rate; FRR, false rejection rate.

**FIGURE 9:**
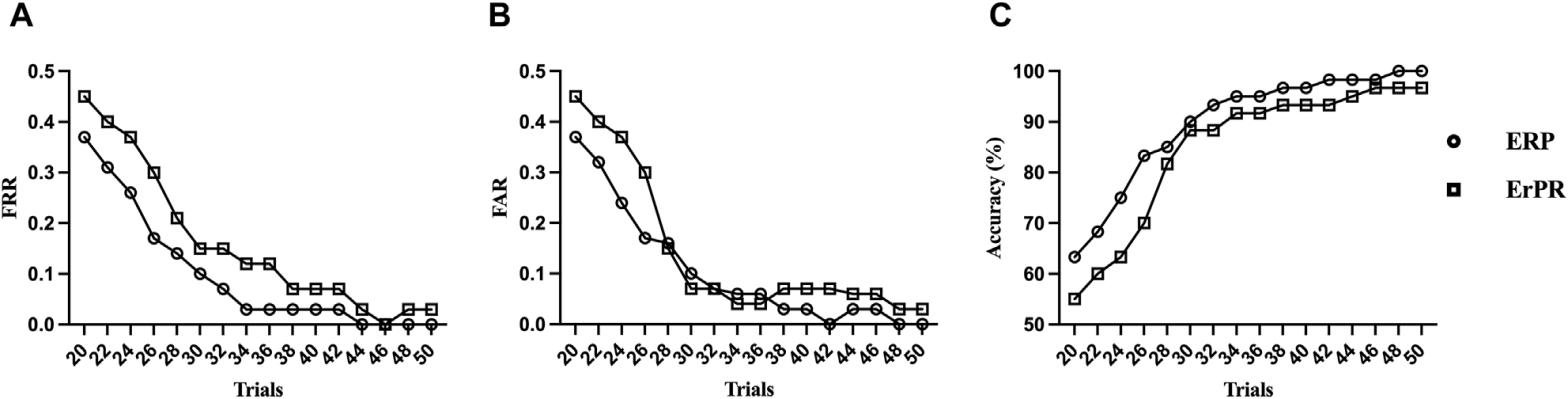
Trends of **(A)** false rejection rate (FRR), **(B)** false acceptance rate (FAR), and **(C)** accuracy by the number of RSVP trials in an ErPR-based authentication system (QDA classifier).

Figure 9 shows the FRR, FAR, and accuracy of the proposed authentication system as functions of the number of trials based on the QDA classifier. The accuracy declines rapidly as the number of trials decreases. The proposed ERP- and ErPR-based authentication systems exhibited accuracies of 95% when the number of trials was less than 34 and 44, respectively. Although the accuracy increases with more tasks, the time required for authentication also increases.

### 3.6 Real-time system for individual identification in AR environment

**FIGURE 10:**
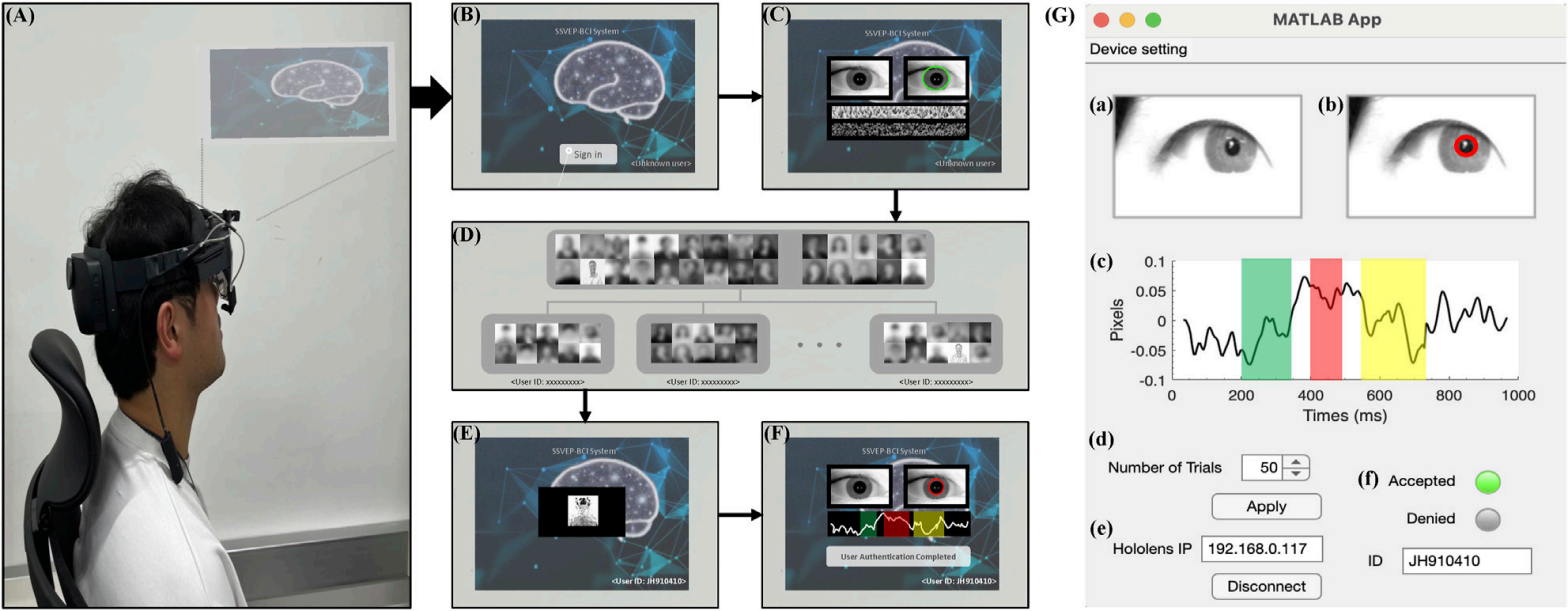
Overview of a real-time system for individual identification in an AR environment. **(A)** Overview of the authentication system in AR environment. **(B)** “Sign in” screen. **(C)** Authentication using iris recognition. **(D)** Database of human photographs. **(E)** ErPR-based authentication tasks. **(F)** Final decision of the proposed authentication system for accessing the target system. **(G)** A real-time system architecture for ErPR-based authentication system: (a) Input infrared image; (b) Detecting pupil area and measuring pupil size; (c) ErPR epoch; (d) Controlling trials of authentication task; (e) Setting IP connection between the AR device and analysis PC; (f) Final decision of individual identification.

The real-time system proposed in this study consists of a Microsoft HoloLens 2 (Microsoft Corp., Redmond, WA, United States), Pupil Labs’ AR binocular eye-tracker (Pupil Labs, Berlin, BB, Germany) add-ons for the HoloLens 2, and a personal computer for analysis. As shown in Figure 10, the target system could be accessed using two-factor individual identification in AR environment. The procedure of individual identification to access the target system is as follows: 1) The user wears the Microsoft HoloLens 2 headset and operates the target system (Figure 10A). 2) Then, the user uses the “Sign in” button to attempt authentication to access the target system (Figure 10B). 3) The proposed system conducts primary authentication by analyzing the user’s iris pattern (Figure 10C). Iris recognition is developed using publicly available open-source code from GitHub (https://github.com/thuyngch/Iris-Recognition). 4) The database comprises photographs voluntarily registered by users who intend to utilize the authentication system, alongside randomly collected photographs of individuals. A unique identifier resulting from iris recognition is assigned to the user, and a random sequence of photographs is generated using target photographs stored in the database (Figure 10D). 5) The authentication tasks are performed, and the pupil image is measured (Figure 10E). 6) The proposed system conducts authentication by analyzing the user’s ErPR pattern. If the user is the real client, ErPR response occurs in reaction to the target photographs, and the authentication system allows access to the target system (Figure 10F). Figure 10G shows the real-time system architecture for individual identification using ErPR response. The ErPR-based authentication system was developed using the MATLAB App designer (2020b, Mathworks Inc., Natick, MA, United States) and Unity 2018.1 (Unity Technologies, San Francisco, CA, United States). In the two-factor-based real-time identity recognition system, the iris recognition accuracy was 100%, and the results of the ErPR-based authentication system are consistent with those reported in Section 3.5 Classification.

## 4 Discussion

We proposed a novel ErPR-based identity authentication system that uses familiar and stranger human faces for RSVP. We demonstrated that the RSVP stimulus based on facial familiarity elicited distinct ErPR and ERP traits in each user and that the ErPR trait can be utilized as an alternative to the ERP-based authentication system. This study assessed the classification performance, which involves accuracy, AUC, FAR, and FRR, between a target (i.e., familiar face) and nontarget (i.e., stranger face) in our RSVP paradigm for two biometric traits. Additionally, to compare the similarities between them, correlation coefficients and Bland–Altman plots were analyzed.

The average ERP epoch of each subject revealed EP components such as P3a (200–350 ms), P3b (400–490 ms), and LPP (530–750 ms) with significant differences in amplitude, although the latency did not show significant differences between targets and nontargets. P3 and LPP components are well-known representative indicators of cognitive processes. The P3 component can be elicited if the user’s brain fully perceives the stimulus. The LPP component is related to post-processing of consciousness, thus indicating advanced cognition and regulation of information (Zhang R. et al., 2022). These components are elicited by strict protocols, in which low-probability “target stimuli” and high-probability “nontarget stimuli” (i.e., a target rate of 10% or 20%) are mixed (Jijomon and Vinod, 2021). Kaongoen et al. (2017) proposed a two-factor authentication system using P300 ERP responses from photographic stimuli; furthermore, the P300 epochs (i.e., 200–750 ms) in the target condition showed a higher amplitude compared to the nontarget. Zeng et al. (2018) developed an identity authentication system using the RSVP paradigm, including the self-face and non-self-face. They reported significant differences in P3a and P3b amplitudes induced by familiar and unfamiliar photographs. Rathi et al. (2021) proposed an authentication system using P300 speller, consisting of pictures of different object pictures with a 2 × 2 matrix. They found that P300 amplitude in the target condition was significantly larger than that in the nontarget condition. Other studies have demonstrated that the amplitude in P3 and LPP components of ERP in target are significantly larger than those in the nontarget, and our findings are consistent with these studies (Lee et al., 2017; Kim et al., 2018; Sabeti et al., 2020; Rathi et al., 2022). However, this study differs from previous studies in that it uses AR glasses in the authentication systems. AR-glass-based authentication systems can provide users with more flexibility than monitor screens, such as freeing both hands and enabling the use of multiple devices (Uhlmann et al., 2019). The proposed ERP-based authentication system achieved perfect accuracy in terms of FAR and FRR using the LSVM and RBF-SVM classifier (5-fold cross-validation).

The pupillary rhythm (i.e., ErPR)-based authentication system proposed in this study exhibited lower performance than ERP, but achieved high performance in accuracy (97%), FAR (0.03), and FRR (0.03) using the QDA classifier (5-fold cross-validation). Similar to the ERP epoch, the amplitude of the average ErPR epoch of each subject in the target stimulus was significantly larger than that of the nontarget stimuli. The PSC is significantly associated with the brain regions related to cognitive processing, involving locus coeruleus–norepinephrine, posterior and anterior cingulate cortex, paracingulate cortex, orbitofrontal cortex, right anterior insular cortex, dorsal anterior cingulate, basal ganglia, lingual gyrus, and thalamus (Joshi et al., 2016; Larsen and Waters, 2018; DiNuzzo et al., 2019; Ceh et al., 2021; Groot et al., 2021; Mäki-Marttunen, 2021). The neural resource caused by cognition for stimuli is reflected in pupil size via a top-down executive control network in the following steps: 1) Alert, an early component (Pa), 2) acceleration of Pa, and 3) executive control by a prominent late component (Pe) (Geva et al., 2013). Many previous studies have reported a strong correlation between PSC and ERP components in cognitive processing. (1) The pupil dilation response is associated with the amplitude of the P3a component in the top-down control of involuntary orienting of attention (Selezneva and Wetzel, 2022). (2) Pupil dilation is related to the amplitude of LPP during cognitive reappraisal (Strauss et al., 2016). (3) The pupil dilation (i.e., ErPR reinstatement data) caused by arousal-related norepinephrine release related to attention is correlated with stronger EEG α-β desynchronization (i.e., event-related desynchronization) and ERP signals (Dahl et al., 2020). (4) Increasing pupil size has been correlated with the amplitude of the P300 and N400 components in cognitive load (Kuipers and Thierry, 2011; Tao et al., 2019) and cognitive flexibility (Kuipers and Thierry, 2013). In our previous studies, we found that the amplitude of the P3 and LPP components in both ERP and ErPR epochs significantly decreased with increased mental load and showed a strong positive correlation between them (Park and Whang, 2018; Park et al., 2019; Park et al., 2022). In this study, the amplitudes of ERP and ErPR epochs were directly related to each other, based on the results for correlation coefficients (i.e., in the range of 0.452–0.829) and the Bland–Altman plot (i.e., fairly good agreement) between them. We identified sufficient evidence that the ErPR of the pupil rhythm could be utilized as an alternative to ERP in authentication systems. The ErPR-based authentication system, especially in an AR environment (i.e., eye-tracker add-on AR glasses), can provide good usability in a simple, economical, and contactless manner.

## 5 Conclusion

This study aimed to develop an infrared camera-based noncontact authentication system using ErPR epochs obtained from pupillary rhythms in an AR environment. The proposed ErPR-based authentication system achieved high performance but showed lower performance than previous EEG signal-based authentication systems (Kaongoen et al., 2017; Chan et al., 2018; Wu et al., 2018b; Chen et al., 2020; Kasim and Tosun, 2021; Zhao et al., 2021). However, the approach presented in this paper allows noncontact authentication for people without the burden of sensor attachment via low-cost, noninvasive, and easily implemented technologies in an AR environment. Although the time required for authentication and effect of variations in ambient light levels must be improved, the proposed method has considerable potential for use in person-authentication systems. Future studies will attempt to overcome the disadvantages of this study.

## Data Availability

The raw data supporting the conclusions of this article will be made available by the authors, without undue reservation.
